# A Rare Case of Eight-and-a-Half Syndrome Due to a Pontine Ischemic Stroke

**DOI:** 10.7759/cureus.85834

**Published:** 2025-06-12

**Authors:** Pedro L Almeida, Tiago Félix, Rafaela Evangelista, Ana Gomes, Ilídia Carmezim

**Affiliations:** 1 Physical Medicine and Rehabilitation, Unidade Local de Saúde de Viseu Dão-Lafões, Viseu, PRT; 2 Internal Medicine, Unidade Local de Saúde de Viseu Dão-Lafões, Viseu, PRT

**Keywords:** brain stem infarction, ischemic stroke, neurology manifestation, physical medicine and rehabilitation, pontine infarction

## Abstract

Eight-and-a-half syndrome is an uncommon entity characterized by a combination of one-and-a-half syndrome and ipsilateral lower motor neuron facial palsy, typically resulting from a lesion in the dorsal pontine tegmentum. Although the clinical presentation may be striking, early diagnosis can be challenging due to subtle imaging findings in the hyperacute phase.

We report the case of a 77-year-old man with no prior disability who presented to the emergency department (ED) with the sudden onset of headache, dizziness, vomiting, diplopia, dysarthria, and gait instability. Neurological examination revealed left lower motor neuron facial palsy, impaired ocular motility with limited adduction of the left eye, horizontal nystagmus, upbeat and downbeat nystagmus, and severe gait ataxia. The clinical constellation was consistent with eight-and-a-half syndrome. A non-contrast CT scan was unremarkable; however, given the acute presentation, intravenous thrombolysis was administered. An MRI performed later confirmed an ischemic lesion in the dorsal pons. The patient showed early neurological improvement and was discharged with a minimal increase in disability. At the three-month follow-up, he remained independent with partial resolution of cranial nerve deficits.

We present this case to highlight the diagnostic value of detailed clinical examination in identifying uncommon brainstem syndromes, even when initial imaging is inconclusive. Recognition of eight-and-a-half syndrome at presentation can guide timely intervention and support clinical decision-making in the management of posterior circulation strokes. Documenting such cases enhances understanding of neuroanatomical correlations and reinforces the importance of clinical vigilance in acute stroke assessment.

## Introduction

Eight-and-a-half syndrome is a rare neurological syndrome resulting from a lesion in the dorsal pontine tegmentum, typically due to ischemic stroke, demyelination, or brainstem tumors. This syndrome is a combination of one-and-a-half syndrome, which includes an ipsilateral horizontal gaze palsy due to damage to the paramedian pontine reticular formation (PPRF) or abducens nucleus, and internuclear ophthalmoplegia (INO) from medial longitudinal fasciculus (MLF) involvement, plus ipsilateral lower motor neuron (LMN) facial palsy caused by facial nerve nucleus or fascicle damage [1-3].

Clinically, patients present with horizontal gaze palsy in one eye, limited adduction in the other eye, and ipsilateral LMN facial weakness, creating a distinct "eight-and-a-half" clinical profile. This syndrome provides precise localization of pontine lesions and is a key diagnostic indicator of brainstem pathology. Though uncommon, prompt recognition is critical, as early intervention, including thrombolysis in ischemic strokes, can improve outcomes [4, 5].

## Case presentation

Presentation and initial assessment

A 77-year-old male patient with a Modified Rankin Scale score of 0 presented to the emergency department (ED) in February 2022 with a sudden onset of left frontotemporal headache, dizziness, vomiting, diplopia, and dysarthria without aphasia, which began three hours prior to arrival. He had been unable to walk since then. The patient denied any history of head trauma. His medical history included hypertension, dyslipidemia, and gout. His regular medications comprised simvastatin 20 mg, ramipril 2.5 mg, hydrochlorothiazide 12.5 mg, and allopurinol 300 mg daily. He reported no known drug allergies. 

**Figure 1:**
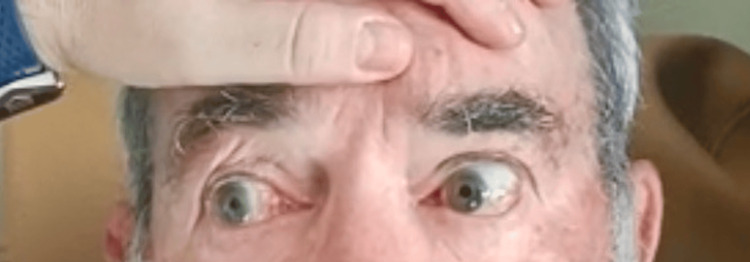
Rightward gaze test; limited adduction of the left eye on rightward gaze is noted. The patient consented to the use of their images in an open-access journal, and a written and signed consent statement from the patient was provided to the journal.

**Figure 2:**
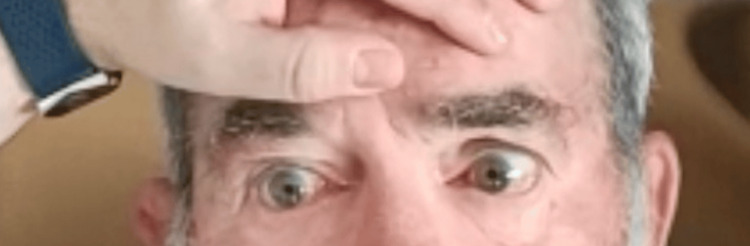
Leftward gaze test; limited adduction of the left eye and impaired abduction of the left eye were noted, which did not reach the midline on leftward gaze. The patient consented to the use of their images in an open-access journal, and a written and signed consent statement from the patient was provided to the journal.

On admission, the patient had slightly elevated arterial pressure (147/72 mmHg) and a sinus cardiac rate of 88 beats per minute (bpm). The neurological examination revealed mild anisocoria with the left pupil slightly larger than the right; left LMN facial nerve palsy with a flattened nasolabial fold, inability to wrinkle the forehead, and incomplete eyelid closure on the left side. Ocular motility testing demonstrated the following limited adduction of the left eye and horizontal nystagmus of the right eye on rightward gaze (Figure 1); limited adduction of the left eye and impaired abduction of the left eye, which did not reach the midline on leftward gaze (Figure 2); upbeat nystagmus on upward gaze; and downbeat nystagmus on downward gaze. Gait ataxia was also present. The rest of the neurological examination was unremarkable, with no other motor, sensory, or language deficits. The patient scored five on the National Institutes of Health Stroke Scale (NIHSS).

An urgent non-contrast head CT scan showed no evidence of an acute cerebrovascular event. However, given the high clinical suspicion for acute ischemic stroke, the ED team proposed treatment with intravenous recombinant tissue plasminogen activator (rTPA) after discussing the risks and benefits with the patient, who consented to the intervention. Thrombolysis was administered without complications.

Hospital course and follow-up

The patient was subsequently admitted to the Stroke Unit with a diagnosis of eight-and-a-half syndrome, suspected to result from an ischemic stroke involving the dorsal pons. The Gugging Swallowing Screen (GUSS) score at admission was 20/20, indicating no significant dysphagia.

**Figure 3:**
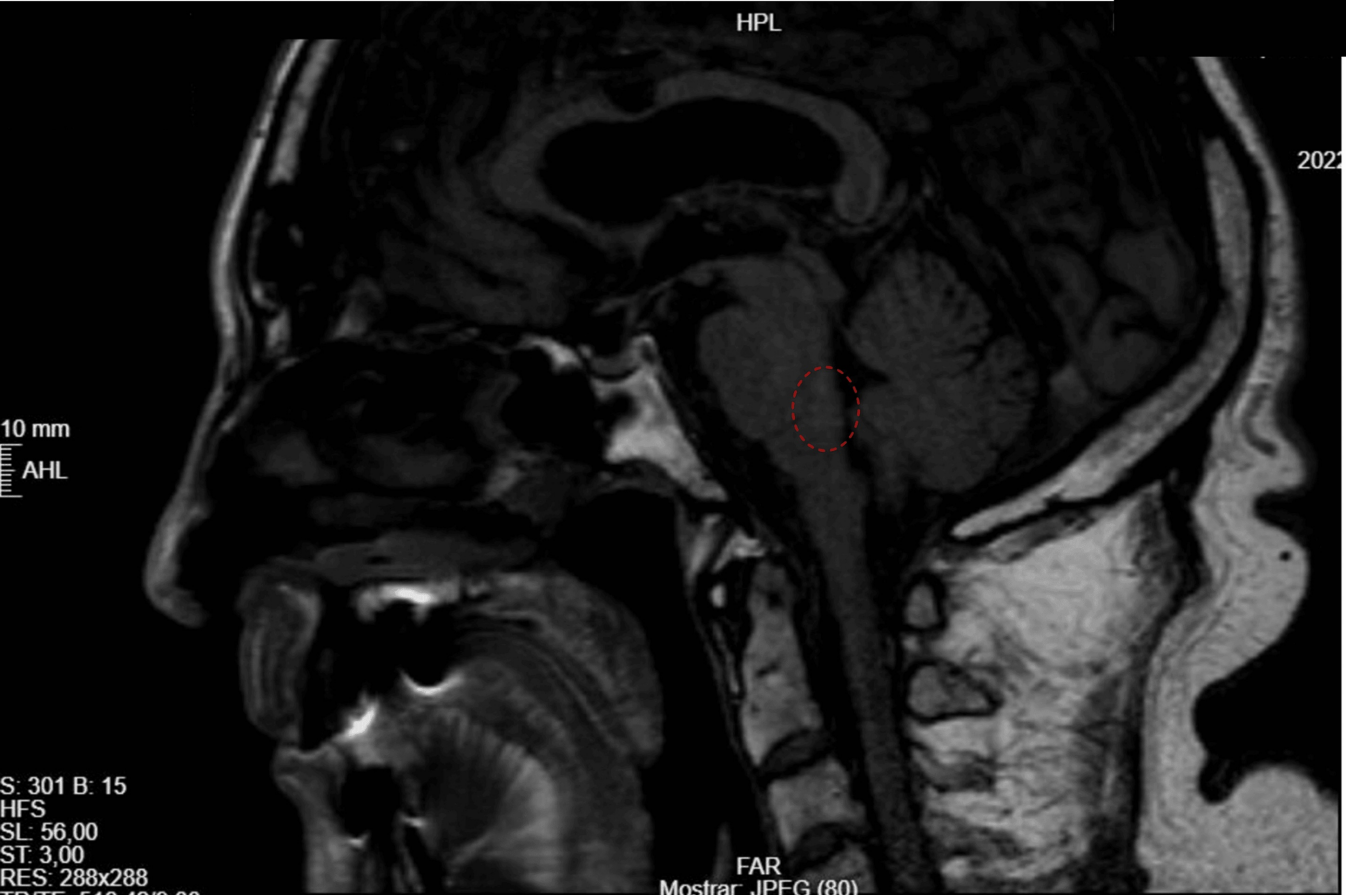
MRI confirming acute ischemic lesion in the dorsal pontine tegmentum (red circle)

A 24-hour post-thrombolysis CT scan showed no ischemic or hemorrhagic lesions. Routine laboratory tests, carotid Doppler ultrasound, transthoracic echocardiogram, and electrocardiogram were unremarkable. However, a subsequent brain magnetic resonance imaging (MRI), which was unavailable at the initial ED evaluation, confirmed an acute ischemic lesion in the dorsal pontine tegmentum, corroborating the suspected diagnosis (Figure 3).

The patient was enrolled in a structured rehabilitation program, including physiotherapy and occupational therapy, and demonstrated early neurological improvements.

After a nine-day hospital stay, the patient was discharged home with a slight increase in his previous Modified Rankin Scale score (0 to one). His secondary stroke prevention regimen was modified, switching simvastatin 20 mg to a combination of rosuvastatin 10 mg and ezetimibe 10 mg daily, along with the initiation of acetylsalicylic acid (aspirin) 100 mg daily. The patient maintained the previously prescribed rehabilitation program in the Physical and Rehabilitation Medicine Unit.

At the three-month follow-up in the Stroke Unit, the patient reported continued participation in physical therapy and showed partial improvement in ocular motility and facial nerve function. He was able to perform daily activities independently.

## Discussion

Eight-and-a-half syndrome: clinical significance

Eight-and-a-half syndrome is a rare neurological condition caused by a lesion affecting the dorsal pontine tegmentum, typically due to an ischemic stroke, demyelinating disease, or brainstem tumors. The syndrome arises from a lesion in the dorsal tegmentum of the caudal pons, specifically affecting critical structures: the abducens nucleus or PPRF, the MLF, and the facial nerve fascicle [6]. The abducens (cranial nerve VI) nucleus/PPRF controls horizontal gaze. A lesion here results in an ipsilateral horizontal gaze palsy. The MLF is a heavily myelinated tract that coordinates conjugate horizontal eye movements by connecting the abducens nucleus to the contralateral oculomotor nucleus. Damage to the MLF leads to INO, characterized by impaired adduction of the ipsilateral eye and nystagmus of the contralateral eye during abduction [7]. The facial (cranial nerve VII) nerve fascicle controls the muscles of facial expression. As the facial nerve fibers loop around the abducens nucleus, a lesion here causes an ipsilateral lower motor neuron facial palsy, affecting both the upper and lower facial muscles [8].

The simultaneous involvement of these structures results in the characteristic clinical presentation of eight-and-a-half syndrome: horizontal gaze palsy with inability to move both eyes horizontally toward the side of the lesion; INO with impaired adduction of the eye on the side of the lesion and nystagmus of the opposite eye during abduction; and facial nerve palsy with weakness of the facial muscles on the same side as the lesion, including the forehead, eyelid closure, and mouth movements. This constellation of deficits leaves the only preserved horizontal eye movement as abduction of the eye contralateral to the lesion [6-9]. The syndrome is named eight-and-a-half because it is essentially the combination of one-and-a-half syndrome (horizontal gaze palsy + INO) and ipsilateral LMN facial palsy.

The most common causes of eight-and-a-half syndrome include vascular events due to ischemic strokes and demyelinating diseases such as multiple sclerosis [6, 7]. Other reported etiologies encompass vascular malformations like cavernous ones that can hemorrhage and compress adjacent neural structures, neoplasms that can directly invade or compress the relevant neural pathways, or infectious diseases such as tuberculosis [8, 10].

Diagnosis is primarily clinical, based on the characteristic findings of horizontal gaze palsy, INO, and facial nerve palsy. Neuroimaging, particularly MRI, is essential to identify the underlying cause, such as infarction, demyelination, or mass lesions. MRI can also help delineate the extent of the lesion and involvement of specific neural structures [9].

The management and prognosis of eight-and-a-half syndrome largely depend on the underlying etiology. Ischemic stroke patients may experience partial or complete recovery over time, depending on the infarct size and promptness of medical intervention [6]. Managing risk factors like hypertension and diabetes, and antiplatelet therapy or anticoagulation may be indicated in secondary prevention for these patients. When caused by demyelinating conditions, treatment with immunomodulatory therapies can lead to improvement, although some deficits may persist [7]. In space-occupying lesions, surgical intervention may be necessary to remove tumors or decompress cavernomas, with variable outcomes based on the lesion’s nature and location [8]. Overall, while some patients may experience significant recovery, others might have persistent deficits, emphasizing the importance of early diagnosis and tailored management strategies.

Rehabilitation strategies

Effective rehabilitation requires a multidisciplinary approach tailored to the individual's specific deficits. It aims to restore independence by leveraging neuroplasticity, the brain’s ability to reorganize neural pathways through repetitive practice. It mainly focuses on ocular motor function and facial nerve recovery [11].

To manage ocular motor function deficits, prism glasses may be used to reduce diplopia (double vision) and assist with eye alignment. Eye movement exercises could be important to enhance the range of motion and improve ocular coordination. In more severe or refractory cases, botulinum toxin therapy injections into extraocular muscles can help reduce strabismus or nystagmus-related symptoms [11].

Facial muscle training and exercises with the use of strengthening exercises intend to activate and retrain facial muscles, and the use of visual feedback strategies helps patients coordinate and enhance facial movements [12, 13]. Patients can also practice controlled facial expressions to improve muscle coordination and reduce synkinesis [14]. Lip and tongue exercises may be important to improve speech articulation [12]. Electrical stimulation therapy with transcutaneous electrical nerve stimulation (TENS) can activate weakened facial muscles, enhancing neuromuscular function and circulation [15]. In cases of post-stroke synkinesis or hypertonicity, botulinum toxin can reduce unwanted muscle contractions and improve facial balance [12].

## Conclusions

Eight-and-a-half syndrome is a rare but distinct neurological condition that allows precise localization of lesions within the pontine tegmentum. Prompt recognition through clinical examination and imaging is crucial for identifying potentially life-threatening causes like infarction or tumors. Understanding the anatomical and clinical nuances of this condition facilitates accurate diagnosis and appropriate management, ultimately improving patient outcomes. While prognosis is generally good for vascular and demyelinating cases, space-occupying lesions require timely intervention. By addressing the diverse impairments resulting from the stroke, rehabilitation aims to maximize functional independence and enhance the quality of life for affected individuals. Early and tailored interventions, considering individual patient factors, are crucial for optimizing recovery outcomes. Comprehensive rehabilitation, including ocular and facial motor therapy, is essential for improving functional outcomes and enhancing the patient's quality of life.
